# RC-LDPC-Polar Codes for Information Reconciliation in Continuous-Variable Quantum Key Distribution

**DOI:** 10.3390/e27101025

**Published:** 2025-09-29

**Authors:** Fei Hua, Kun Chen, Wei Deng, Jing Cheng, Banghong Guo, Huanwen Xie

**Affiliations:** 1 Guangdong Provincial Key Laboratory of Nanophotonic Functional Materials and Devices, School of Optoelectronic Science and Engineering, South China Normal University, Guangzhou 510006, China; huaf666@163.com (F.H.); ck_bsqc@163.com (K.C.); 13049661600@163.com (W.D.); cj13437202110@163.com (J.C.); 2 Guangdong Provincial Key Laboratory of Quantum Engineering and Quantum Materials, School of Optoelectronic Science and Engineering, South China Normal University, Guangzhou 510006, China; 3 National Quantum Communication (Guangdong) Co., Ltd., Guangzhou 510700, China; vp_eng@nqctek.com

**Keywords:** continuous-variable quantum key distribution, cascade polar codes, information reconciliation, rate compatibility

## Abstract

Continuous-variable quantum key distribution faces significant challenges, including quantum channel instability, particularly fluctuations in the signal-to-noise ratio (SNR) and extremely low SNR scenarios. Furthermore, non-ideal polar codes, characterized by insufficient polarization in finite-length regimes, can lead to some sub-channels being neither completely noise-free nor fully noise-dominated. This phenomenon limits the error correction capability when such codes are applied to information reconciliation. To address these challenges, we propose a novel RC-LDPC-Polar code for the CV-QKD reconciliation algorithm. We combine the error resilience of LDPC codes with the efficiency advantages of polar coding. This scheme supports adaptive rate adjustment across varying SNR conditions. Our simulation experiments demonstrate that the RC-LDPC-Polar concatenated coding scheme achieves a lower error rate under varying SNR conditions. Meanwhile, the proposed scheme achieves a higher final key rate and a longer transmission distance.

## 1. Introduction

Quantum key distribution (QKD) enables unconditionally secure communication when combined with one-time pad (OTP) encryption [1]. In Continuous-variable quantum key distribution (CV-QKD), the Gaussian-modulated coherent-state CV-QKD protocol provides proven security [2]. This protocol requires communicating parties (Alice and Bob) to share correlated Gaussian variables for post-processing key extraction. Researchers have developed two primary negotiation approaches: slice negotiation [3] and multi-dimensional negotiation [4]. Slice negotiation performs optimally in high-SNR conditions, while multi-dimensional negotiation maintains efficiency in low-SNR, long-distance scenarios. Polar codes represent a class of capacity-achieving error-correcting codes [5]. Their Shannon-limit attainment makes them particularly suitable for CV-QKD information reconciliation [6,7,8,9].

The quantum channel’s dynamic SNR variations demand careful consideration. Fan et al. developed an LDPC-based rate-adaptive reconciliation scheme [10] for fluctuating channels. The scheme achieved efficient error correction under channel variations. However, at very low SNR (corresponding to long-distance QKD), LDPC codes exhibit an “error floor” phenomenon, limiting the maximum transmission distance. Zhou et al. introduced a Raptor-like LDPC code-based rate-compatible scheme [11], which mimics the rateless concept of Raptor codes. It still fails to overcome the error floor issue inherent in LDPC codes, although it optimizes the degree distribution within the LDPC framework. Wang et al. proposed flexibly adjusting the code rate by randomly inserting punctured and shortened bits [12]. Yet, this approach cannot guarantee optimal degree distribution or Hamming distance after each operation, thus failing to achieve the best possible error correction performance for the given code length.

Finite-length polar codes for information reconciliation also demand careful consideration. Practical finite-length polar codes are highly sensitive to dynamic channel variations, although polar codes can approach the Shannon limit under ideal conditions. Their performance heavily relies on accurate matching with channel states; rate mismatch can significantly reduce reconciliation efficiency or even cause complete decoding failure. To enhance the adaptability of polar codes, Wang R introduced a generating matrix shortening method [13] that removes specific rows and columns with unit column weight. The puncturing (QUP) method [14] is simple to implement and exhibits stable performance with the minimum row weight property. Both methods, however, neglect Hamming distance optimization in rate-compatible designs, limiting their performance in multi-rate scenarios. Li et al. [15] indicated that constructing polar codes with a larger Hamming distance can improve performance. Therefore, designing efficient rate-compatible polar codes by combining channel reliability and Hamming distance constraints is feasible. Some scholars [15,16,17,18,19,20] have proposed concatenated coding schemes that combine polar codes with other error-correcting codes (ECCs). LDPC codes [21] serve as inner or outer codes [22] in serial concatenation with polar codes. This architecture leverages LDPC’s superior error correction for medium-length polar codes while utilizing the error floor mitigation properties of polar codes. Partial serial cascading [23,24] further optimizes efficiency by selectively precoding intermediate subchannel codewords.

This paper constructs rate-compatible LDPC-Polar (RC-LDPC-Polar) codes for CV-QKD information reconciliation. The proposed method applies matrix shortening to polar codes. The shortening process follows two key principles: column weight of 1 and row weight minimization. This adaptation maintains performance under channel variations. We implement LDPC precoding on intermediate subchannels of the rate-compatible polar codes. Simulation results demonstrate that the proposed RC-LDPC-Polar cascade code achieves a lower bit error rate (BER). The proposed scheme exhibits superior reconciliation efficiency and higher decoding speed compared with existing protocols of information reconciliation [6,11,25,26].

The rest of the paper is organized as follows. In Section 2, the information reconciliation method with RC-LDPC-Polar codes is described. Section 3 explains the construction method of RC-LDPC-Polar codes. Section 4 provides simulation results and compares the proposed scheme with existing approaches. Finally, conclusions are drawn in Section 5.

## 2. RC-LDPC-Polar Code-Based Reconciliation

**Figure 1:**
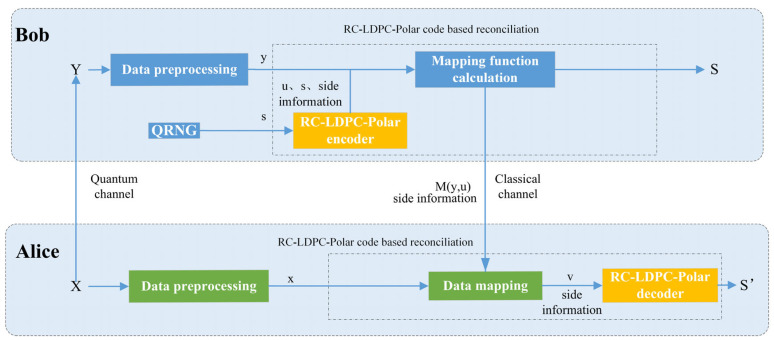
Multidimensional reconciliation scheme for RC-LDPC-Polar codes. X, Y: Correlated Gaussian variables held by Alice and Bob, respectively, where Y=tX+Z. s: Secret binary sequence generated by Bob using a quantum random number generator (QRNG). c: Encoded codeword sequence, output by the RC-LDPC-Polar encoder. u: d-dimensional spherical vector mapped from the codeword c. $M\left(y,u\right)$: Rotation mapping function calculated by Bob, satisfying $M\left(y,u\right)\cdot y=u$. v: Noisy version of the sequence c obtained by Alice after applying the rotation mapping $M\left(y,u\right)$ to her variable X. $s^{\prime }$: Estimated secret sequence decoded by Alice using the RC-LDPC-Polar joint BP decoder.

This study combines RC-LDPC-Polar codes with reverse multidimensional reconciliation [27] in variable SNR channels, as illustrated in Figure 1. Communicating parties establish a binary-input additive white Gaussian noise (BI-AWGN) virtual channel through Gaussian variable rotation. Sequence discrepancies are subsequently corrected using the proposed RC-LDPC-Polar coding scheme. The core procedure comprises three key steps: (i) encoding and mapping at the Bob side; (ii) information transmission over the quantum and classical channels; and (iii) joint decoding at the Alice side. By leveraging the powerful error correction capability of the LDPC-Polar concatenated code and the multidimensional reverse reconciliation, the proposed scheme effectively addresses the challenge of dynamic SNR fluctuations.

We explain the process of the scheme in detail. Alice and Bob hold initially Gaussian-distributed key vectors X and Y (dimension n), where Y=tX+Z. The parameter t denotes channel loss, and Z represents Gaussian noise. Both parties first preprocess their vectors. Alice and Bob divide the initial keys X and Y into d-dimensional vectors (d=8 in our scheme), and normalize them into x and y, respectively.

As the reconciler, Bob generates a random binary sequence s using a quantum random number generator. This sequence is encoded by the RC-LDPC-Polar encoder into a sequence c. Subsequently, c is converted to a d-dimensional spherical vector u, $u\in {\left\{\frac{-1}{\sqrt{d}},\frac{1}{\sqrt{d}}\right\}}^{d}$.

Bob computes the rotation mapping function $M\left(y,u\right)$ satisfying $M\left(y,u\right)\cdot y=u$. Bob sends the mapping function $M\left(y,u\right)$ along with side information to Alice, through a publicly authenticated channel. Alice uses this function to map a sequence x to v such that $v=M\left(y,u\right)\cdot x$. The sequence v is a noisy version of c. Then Alice performs RC-LDPC-Polar joint BP decoding on v and output the estimated sequence $s^{\prime }$, using the side information. Alice and Bob obtain consistent secret keys if the decoding is successful. And Bob generates more mapping function $M\left(y,u\right)$ to initiate the next negotiation round.

The CV-QKD system key rate is determined by both the optical system and the post-processing, and is expressed in terms of the key rate K as:

(1) $$K=\frac{n}{N}\cdot (1-FER)\cdot (\beta I_{AB}-\chi_{BE}-\Delta (n)),$$

in ideal conditions, the asymptotic secret key rate of a CV-QKD system can be expressed as [28]:

(2) $$K_{ideal}=I_{AB}-\chi_{BE},$$

where n and N are the number of codewords there are for key extraction and the number of codewords used for data coordination, respectively, determined by the post-processing part. FER is the frame error rate. β is reconciliation efficiency; $I_{AB}$ and $\chi_{BE}$ are the amount of mutual information and the Holevo boundaries of Alice and Bob on the two sides of the communication, respectively, determined by the optical system; and $\Delta \left(n\right)$ is the secrecy-enhancing offset factor.

From Equation (1), FER and β affect the generation of the system security key rate, and β is defined as follows:

(3) β=RC,

where C is the binary input additive Gaussian white noise (BI-AWGN) channel capacity, $C=\frac{1}{2}\log_{2}\left(1+SNR\right)$. R is the code rate of the RC-LDPC-Polar code, R≤C, and the value of β is in the range [0, 1].

## 3. Construction for RC-LDPC-Polar Codes

This section describes the construction process, including polar code shortening and LDPC-polar concatenation for improved error correction. This section elaborates on the construction method of the RC-LDPC-Polar code. First, we construct a rate-compatible polar code using an optimized shortening method (Section 3.1). Then, the subchannels are classified according to their reliability to identify those requiring LDPC precoding (Section 3.2). Finally, the entire codeword is formed via serial concatenation of the component codes (Section 3.3).

### 3.1. Rate-Compatible Polar Code Construction via Shortening

We propose a rate-compatible polar code construction algorithm combining generating matrix operations with Reed-Muller (RM) code optimization. The method initially establishes the polar code structure via a Gaussian approximation [29], then incorporates RM code principles [30] to minimize Hamming distance during shortening. A matrix-based shortening method [13] subsequently enables dynamic rate adaptation. The specific steps are as follows:

Step 1: Original construction: The communication parties first calculate the optimal code rate using the quantum channel’s signal-to-noise ratio (SNR). They determine the target code length M for RC-LDPC-Polar codes and derive the original polar code length N. Through Gaussian approximation, the system obtains subchannel reliability metrics, channels, and constructs the initial generator matrix $G_{N}$.

Step 2: Shortening construction: The algorithm initializes a shortening pattern P as a 1 × N zero vector and computes the shortening bits $N_{p}=N-M$. After sorting subchannel reliability values in descending order, it selects shortening positions in $G_{N}$ using a two-stage criterion: first identifying column-weight-1 positions in $G_{N}$, then choosing among these the subchannels with minimal row weights. When row weights are equal, the system selects the least reliable subchannel using Gaussian approximation. The corresponding bits in P are set to 1, and $G_{N}$ is reduced to ${G_{N}}^{\prime }$ by removing the selected rows and columns. This helps mitigate the performance impact of redundancy removal operations [13] and preserves the distance properties of polar codes [31], thereby enhancing error correction capabilities.

Step 3: Frozen bit allocation: the algorithm identifies the remaining frozen bit positions by selecting subchannel indices in the shortened generator matrix ${G_{N}}^{\prime }$. The positions simultaneously satisfy two conditions: minimal row weight and lowest reliability value among all candidate positions.

Step 4: Information bit allocation: The algorithm partitions the remaining channel indices into two classes:

- Intermediate channels (k_inter_index): this set comprises the first LDPC_N least reliable indices. These channels will carry bits that are pre-coded by the LDPC code.
- High-reliability channels (k_good_index): the remaining indices are used for uncoded information transmission.

### 3.2. Cascaded Construction of RC-LDPC-Polar Code

We define a partially serial iterative rate-compatible cascade code as follows: given two linear block codes $U_{0}$, $U_{1}$ with code length parameters $\left(N_{0},K_{0}\right)$ and $\left(N_{1},K_{1},N_{p}\right)$. A partially serial cascade code $S=\left\{U_{0},U_{1,I^{C}}\right\}$ can be constructed, with code length parameters $\left(N_{s},K_{s}\right)$, where $N_{p}$ is the number of shortened bits, denoting the set of indexes of the $I^{C}$ unreliable subchannels. The actual code length of the serial iterative rate-compatible cascade code $N_{s}=N_{1}-N_{p}$, the information code length is $K_{s}=K_{0}+K_{1}-N_{0}$, and the actual code rate of the cascade is Rs=KsNs.

**Figure 2:**
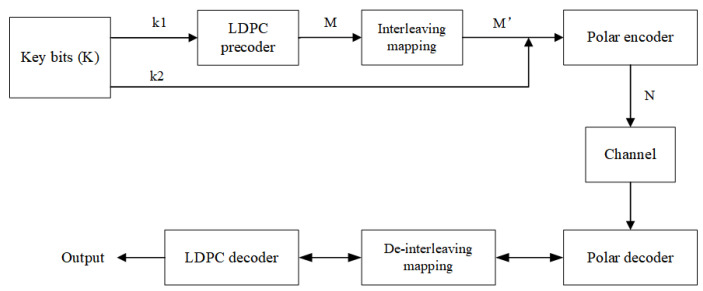
Improved serial cascade structure of the LDPC-Polar section. K: information bits; $k_{1}$: bits for direct encoding; $k_{2}$: bits requiring LDPC precoding; M: LDPC-encoded bits; $M^{\prime }$: interleaved bits; N: polar-encoded codeword.

Figure 2 illustrates the LDPC-Polar partial serial concatenation structure, where LDPC codes serve as outer codes and polar codes as inner codes. This architecture specifically pre-codes information bits transmitted through polar intermediate channels and enables bidirectional information exchange during decoding. The intermediate channel selection critically impacts the system’s BER performance. Unlike traditional schemes based on Bhattacharyya parameters with threshold limitations and BP decoding incompatibility, we formulate channel selection as a leaf-set capacity minimization problem [31]. For further optimization, an interleaved bit mapper is added to the cascade structure, thus maximizing the dispersion of burst errors and controlling the flow of information between the decoders. This provides customized external information based on the polarization sub-channel log-likelihood ratio (LLR) differences.

**Figure 3:**
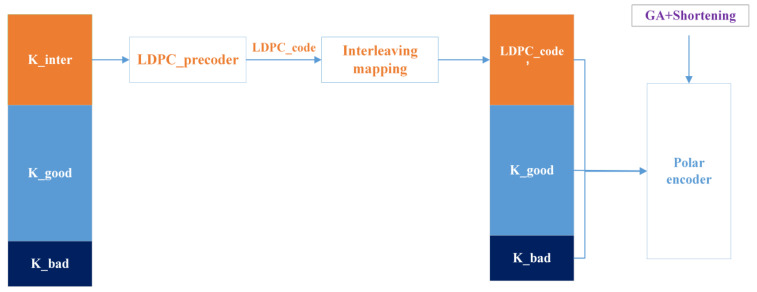
Schematic diagram of RC-LDPC-Polar code construction. After reliability ordering Via Gaussian approximation, polarized subchannels are classified into three categories: high-reliability ($C_{good}$), intermediate-reliability ($C_{\mathrm{int}er}$), and low-reliability ($C_{bad}$) channels. $K_{\_good}$ bits are transmitted directly, $K_{\_\mathrm{int}er}$ bits are LDPC-precoded before being assigned to $C_{\mathrm{int}er}$ channels, and $K_{\_bad}$ positions are fixed as frozen bits.

Building on the channel indexing from Section 3.1’s shortening construction, we implement a partial serial LDPC cascade, as shown in Figure 3. Using Gaussian approximation, we classify polarized channels into three reliability categories: high-reliability $C_{good}$, intermediate $C_{\mathrm{int}er}$, and low-reliability $C_{bad}$ channels.

(4) $$C_{good}=\{W\left(i\right):C\left(i\right)>\delta_{2}\},$$

(5) $$C_{\mathrm{int}er}=\{W\left(i\right):\delta_{1}<C\left(i\right)<\delta_{2}\},$$

(6) $$C_{bad}=\{W\left(i\right):C\left(i\right)<\delta_{1}\},$$

where W(i) denotes the *i* th sub-channel, $i\in \left[1,N\right]$; $C\left(i\right)$ denotes the reliability value of the i th sub-channel; $\delta_{1}$ and $\delta_{2}$ are two real numbers satisfying $\delta_{2}>\delta_{1}>0$.

Key information bits K (length is L) are partitioned into directly encoded bits K_good and doubly encoded bits K_inter (i.e., K={K_good,K_inter}), with randomly generated frozen bits K_bad(length is $\left(N_{s}-L\right)$). During channel mapping, K_good and K_bad are transmitted through a high-reliability channel $C_{good}$ and a low-reliability channel $C_{bad}$ respectively, while K_inter undergoes LDPC encoding to produce parity-check codeword LDPC_code. The resulting LDPC_code′ is transmitted via an intermediate-reliability channel $C_{\mathrm{int}er}$, after bit-interleaving optimization. The concatenated sequence {LDPC_code′,K_good} is then combined with K_bad as input to the polar encoder, ultimately generating a serially concatenated RC-LDPC-Polar code with code length $N_{s}$ and code rate Rs=LNs.

### 3.3. Error Correction System Model

**Figure 4:**
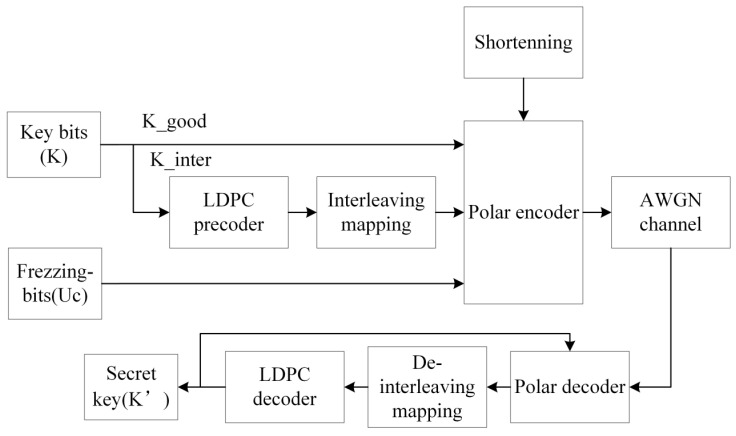
Model of error correction system based on RC-LDPC-Polar code. Transmitter: information bits undergo LDPC precoding, bit interleaving, and polar encoding, followed by shortening to produce the final codeword. Receiver: the received signals are processed by an iterative joint BP decoder to recover the transmitted information. Solid arrows indicate the main data flow.

Multi-dimensional reconciliation includes two steps: error correction and multi-dimensional negotiation. We propose an error correction system model based on RC-LDPC-Polar codes as shown in Figure 4, where LDPC codes are constructed using random construction, PEG construction, and QC-LDPC codes [10]. LDPC codes employ a QC structure, facilitating hardware implementation and ensuring compatibility with the BP decoders of polar codes.

In the transmitter, a k-bit original key bit is divided into two parts of key bits, i.e., key bits K_good, K_inter with lengths k_1_ and k_2_, respectively. The K_good transmits directly via high-reliability polarized channels, while K_inter undergoes LDPC precoding for intermediate-channel transmission. Following LDPC encoding and bit-interleaving, the resulting LDPC_code combines with K_good and frozen bits Uc for polar encoding. The N-bit output undergoes dynamic shortening to M bits, generating the final RC-LDPC-Polar code optimized for SNR adaptation. And we use joint BP decoding of polar code and LDPC code to obtain the estimated original key bit K′. The RC-LDPC-Polar code is co-optimized with the encoding architecture, leveraging RM code row-weight constraints to maximize Hamming distance—a key distinction from traditional shortening methods.

## 4. Simulation Experiments and Discussion

In this section, we evaluate the information reconciliation performance based on the proposed RC-LDPC-Polar code. We analyze the performance of RC-LDPC-Polar codes under low SNR and unstable channels, the reconciliation efficiency, and the key rate. In addition, we use the BP joint decoding method, and the number of iterations of LDPC is taken as 1.

**Figure 5:**
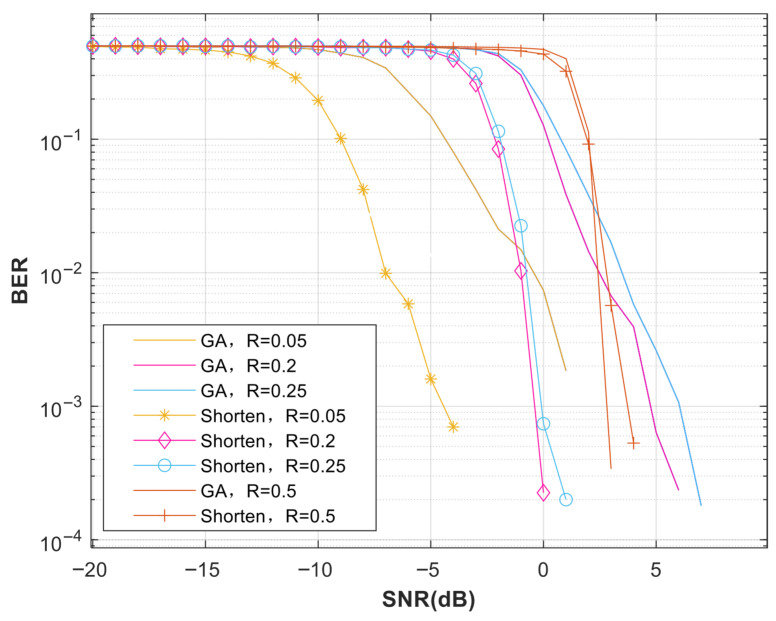
Bit error rate (BER) comparison between the proposed shortening method and the conventional Gaussian Approximation (GA) construction at different code rates. The total code length of the polar code is N = 512, the actual code length after shortening is M = 400, and the code rate of the polar code is 0.05, 0.2, and 0.25, respectively.

Figure 5 compares the BER performance between the conventional Gaussian approximation construction and our proposed shortening method under identical SNR conditions. Experimental results demonstrate a lower bit error rate (BER) for the proposed shortening method compared to conventional constructions. This improvement directly results from our distance-preserving shortening criteria. The method minimizes row weight and retains unit column weight. It thus overcomes the Hamming distance degradation typical in random puncturing schemes [12,14]. A significant gain of approximately 6 dB is observed at a BER of 0.01 for low code rates.

**Figure 6:**
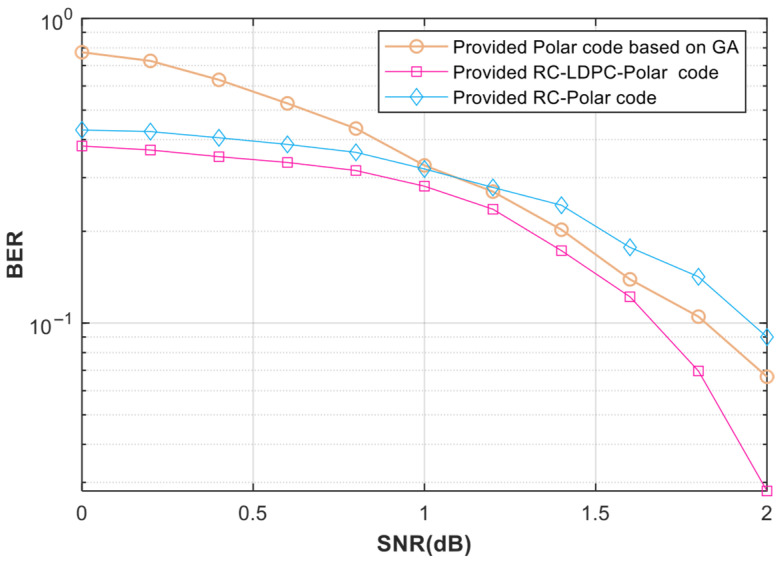
BER under different code lengths and different signal-to-noise ratios. The Polar code’s length N = 256 and code rate of 0.5. The RC-LDPC-Polar code and the RC-Polar code have an actual code length M = 250 and an equivalent code rate of 0.5.

**Figure 7:**
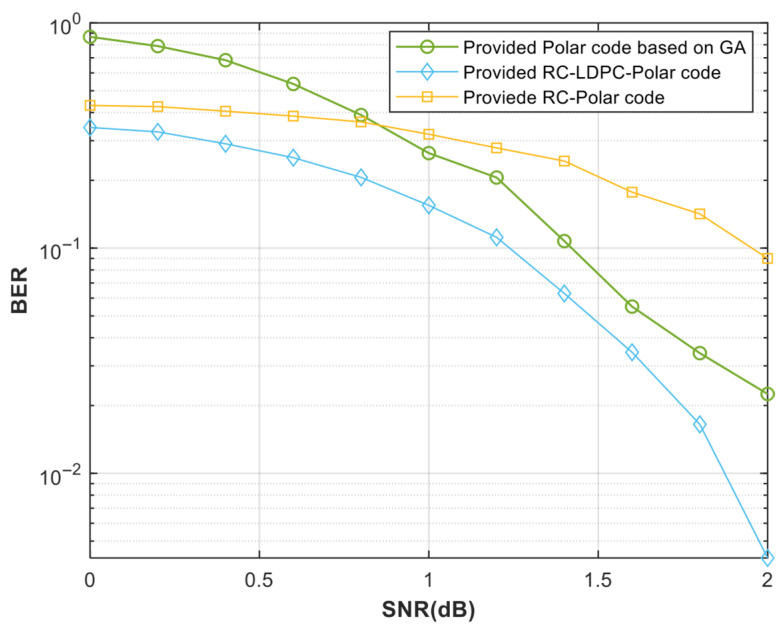
BER under different code lengths and different signal-to-noise ratios. The Polar code’s length N = 512 and code rate of 0.5. The RC-LDPC-Polar code and the RC-Polar code have an actual code length M = 500 and an equivalent code rate of 0.5.

Figure 6 and Figure 7 show that the RC-LDPC-Polar code outperforms both pure polar and rate-compatible polar codes across various SNRs. This superiority arises from the complementary structure of the concatenated design. The LDPC precoder enhances error correction on intermediate channels. It compensates for the insufficient polarization in finite-length polar codes. Simultaneously, the polar code suppresses the error floor inherent in LDPC codes [10,11]. The scheme also avoids the performance loss seen in merely shortened polar codes without auxiliary coding.

These results confirm that our design effectively addresses key limitations of prior methods. The optimized shortening ensures stable performance across rates, and the concatenated structure provides robustness in low-SNR conditions. Thus, the observed improvements are direct consequences of our targeted architectural and algorithmic innovations.

Reconciliation efficiency and key rate are important indicators of information reconciliation capacity. Table 1 compares the reconciliation performance of different error codes, and the actual SNR of the system is around 0.02. The proposed RC-LDPC-Polar code achieves superior performance compared to existing MET-LDPC [25] and RL-LDPC [26] codes at a fixed code length of 10^6^, with higher coordination efficiency, lower FER, and a decoding speed of 21.96 Mbit/s.

**Figure 8:**
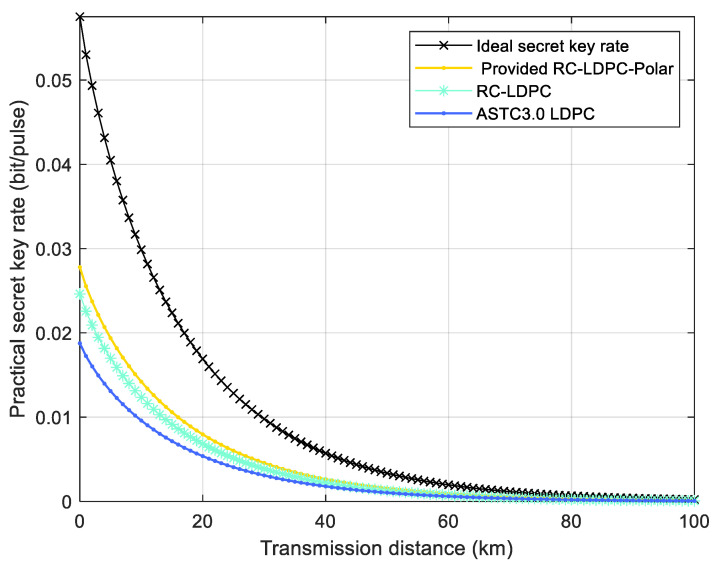
Actual secret key rate vs. transmission distance. The extra noise is 0.01, the detection efficiency is 0.64, and the electronic noise is 0.1.

Figure 8 compares the actual key rates of three coding schemes: the proposed RC-LDPC-Polar code, RC-LDPC [10], and ATSC3.0 LDPC code [10]. Our implementation employs multidimensional coordination to reverse the conventional sequence of parameter estimation and information reconciliation. The results demonstrate that the RC-LDPC-Polar code maintains superior secure key rates compared to IC-LDPC Polar codes across transmission distances of 0–100 km. It can be seen that the RC-LDPC-Polar code is more advantageous when applied to the CV-QKD system.

## 5. Conclusions

In this paper, we propose an RC-LDPC-Polar code for CV-QKD reconciliation to address fluctuating and low SNR conditions in quantum channels. The RC-LDPC-Polar code combines rate-compatible polar codes with LDPC concatenation. We establish an error correction system incorporating multidimensional reconciliation based on RC-LDPC-Polar codes. The main contribution of this work is threefold: (i) A refined shortening construction for polar codes that prioritizes both unit column weight and minimal row weight, better preserving the Hamming distance and enabling more effective rate adaptation compared to conventional puncturing or shortening methods. (ii) A partial serial concatenation scheme where LDPC codes are selectively used to pre-code bits assigned to the intermediate-reliability subchannels of the polarized channel, effectively combining the error correction strength of LDPC with the capacity-achieving potential of polar codes. (iii) The integration of this code into a multidimensional reconciliation protocol, creating a system highly resilient to channel variations. Simulation results demonstrate that this approach successfully addresses the core research question. The proposed scheme provides a definitive performance improvement, achieving a lower error rate across a wide SNR range (particularly between 0 and 2 dB), a higher reconciliation efficiency of 98.07%, and a faster decoding speed of 21.96 Mbit/s at an ultra-low SNR of 0.02, compared to existing state-of-the-art protocols. Meanwhile, our solution achieves a higher secret key rate compared to other schemes.

For long code lengths, the construction complexity of the RC-LDPC-Polar code, as well as the complexity of the joint decoding algorithm, will affect the decoding performance and speed of the concatenated code. In future work, deep learning-assisted scheduling may be considered to optimize the decoding algorithm, reduce computational complexity, and improve system performance.

## Figures and Tables

**Table 1:** Reconciliation performance for different error codes.

Code Type	Block Length	Implementation Platform	SNR	R	β	FER	Max Iterations	Decoding Speed (Mbit/s)
Polar [6]	2^27^	CPU	0.16	-	92.80%	0.090	-	7.3
MET-LDPC [25]	10^6^	GPU	0.029	0.02	96.99%	0.375	200	14.00
RL-LDPC [11]	10^6^	GPU	0.022	0.02	96.00%	0.453	200	16.41
QC-MET-LDPC [26]	10^6^	GPU	-	0.02	99.00%	0.883	500	-
RC-LDPC-Polar	10^6^	FPGA	0.02	0.02	98.07%	0.639	250	21.96

## Data Availability

Data are contained within the article.
